# Bioactive Compounds and Antioxidant Composition of Nut Bars with Addition of Various Edible Insect Flours

**DOI:** 10.3390/molecules28083556

**Published:** 2023-04-18

**Authors:** Dorota Gumul, Joanna Oracz, Stanisław Kowalski, Anna Mikulec, Magdalena Skotnicka, Kaja Karwowska, Anna Areczuk

**Affiliations:** 1 Department of Carbohydrate Technology and Cereal Processing, Faculty of Food Technology, University of Agriculture in Krakow, 122 Balicka Street, 30-149 Krakow, Poland; 2 Institute of Food Technology and Analysis, Faculty of Biotechnology and Food Sciences, Lodz University of Technology, 2/22 Stefanowskiego Street, 90-537 Lodz, Poland; 3 Faculty of Engineering Sciences, University of Applied Science in Nowy Sacz, 1a Zamenhofa Street, 33-300 Nowy Sacz, Poland; 4 Department of Commodity Science, Faculty of Health Sciences, Medical University of Gdansk, 80-210 Gdansk, Poland

**Keywords:** edible insects, nut bar, phenolic compounds, tocopherols, phytosterols, antioxidant properties

## Abstract

Edible insects represent a new functional source of nutrients that can contribute to solving nutritional deficiency problems. The antioxidant potential and bioactive compounds of nut bars with the addition of three edible insects were evaluated. *Acheta domesticus* L., *Alphitobius diaperinus* P. and *Tenebrio molitor* L. flours were used. A 30% share of insect flour in the bars resulted in significantly greater antioxidant activity (TPC increased from 190.19 for standard bars to 309.45 mg catechin/100 g for bars with 30% addition of cricket flour). Insect flour contributed significantly to an increase in 2,5-dihydrobenzoic acid (from 0.12 for bars with a 15% share of buffalo worm flour to 0.44 mg/100 g in the case of bars with a 30% share of cricket flour) and chlorogenic acid in all bars (from 0.58 for bars with a 15% share of cricket flour to 3.28 mg/100 g for bars with a 30% addition of buffalo worm flour), compared to the standard. The highest content of tocopherols was found in bars with cricket flour, compared to standard bars (43.57 and 24.06 mg/100 g of fat, respectively). The dominant sterol in bars enriched with insect powder was cholesterol. The highest amount of it was found in cricket bars, and the lowest in mealworm bars (64.16 and 21.62 mg/100 g of fat, respectively). The enrichment of nut bars with insect flours raises the levels of valuable phytosterols in the final product. The addition of edible insect flours reduced the perception of most sensory attributes of the bars, compared to the standard bar.

## 1. Introduction

The global snack market is characterized by continued growth, and is forecast to grow annually by about 2.7% until 2030. While a lifestyle change of eating smaller meals has been observed over the past few years, it has also contributed to the increased consumption of snacks. This is why snack bars, especially those with protein or functional additives, are in growing demand, especially among physically active people. The change in consumer behavior, including the abandonment of traditional animal protein, is primarily due to people’s growing concerns about animal welfare or sustainability. Consumers are increasingly looking for products that will help them maintain a healthy and balanced diet without compromising the aforementioned factors [1]. It is important to look for alternatives to meet people’s nutritional needs should traditional production prove to be severely compromised, or even impossible. Therefore, a good solution might be to enrich popular snacks such as peanut bars with raw materials that are a good source not only of protein with high biological value, but also a number of valuable nutrients such as vitamins, minerals, and bioactive compounds. The edible insects most often used for research on inclusion in food production, and at the same time approved in the EU for consumption [2,3], are the yellow mealworm (*Tenebrio molitor* L.), buffalo worm (*Alphitobius diaperinus* P.) and the house cricket (*Acheta domestica* L.). These insects have already been used to enrich a number of food products, including bread [4,5,6,7,8], gluten-free bread [9], biscuits [10], muffins [11], pancakes [12] snacks [13], extrudates [14], bars [15] and as a meat substitute in hamburgers [16].

Insects constitute about 80% of the animal kingdom [17]. In Africa, Asia, Central America, and South America, insects are regarded as a good alternative source of vitamins, minerals, proteins, fats, and calories on a yearly basis. Insects contain amino acids, such as methionine, cysteine, lysine, and threonine. The essential amino acid contents of edible insects range from 10% to 30%, while all the amino acid contents range from 35% to 50%. Additionally, their protein digestibility, usually after removing the exoskeleton, was found to be 77–98%. Mineral element analyses revealed that edible insects are rich sources of phosphorus, calcium, manganese, copper, zinc, sodium, potassium, and iron. Edible insects are believed to be good sources of carotene and vitamins, such as B1, B2, B6, D, E, K, and C [18,19,20]. Edible insects contain up to 20% sugars and fatty acids [21].

In recent years, attention has also been paid to the potential for edible insects to be not only a source of nutrients, but also of bioactive compounds, including phenolic compounds, tocopherols and phytosterols. However, studies on the contents of these bioactive compounds, especially phenolic compounds, are still scarce. So far, the focus has been restricted to the nutritional value of edible insects. Phenolic compounds are secondary plant metabolites characterized by the presence of one or more aromatic rings possessing at least one hydroxyl group attached to the aromatic structure. Extensive research shows that phenolic compounds have several bioactivities, including antioxidant, anti-inflammatory, antimicrobial, and anticancer activities [22,23,24,25], among others, and diets rich in phenolics are associated with human health benefits [26]. Phenolic compounds are incorporated into edible insects by a process called sclerotization, in which the insect’s epidermis hardens as a result of a series of enzymatic reactions integrating plant polyphenols from the insect’s diet into the parent epidermis, including structural proteins and chitin [27]. The insect sheds its shell periodically, so the above process will be repeated multiple times in the insect’s lifecycle. In addition, it has been reported that the phenolic content in an insect will be closely related to its diet [26,27]. Research also shows the presence of phenolic compounds in insects (mainly butterflies) as a result of the absorption of dietary phenols, as well as their ability to metabolize these compounds and incorporate them into their structure. Interestingly, insects appear to be capable of the selective absorption of flavonols, mainly kaempferol and quercetin, as well as flavones such as tricin and isovitexin. Most of these are glycosylated with only one compound, i.e., glucose, rhamnose or galactose. Both flavonols and flavones are synthesized by the host plant and are later metabolized or absorbed by the insect [28,29,30]. Edible insects may also be a rich source of sterols comprising mostly phytosterols [31,32]. The study of Cheseto et al. (2015) demonstrated that desert locusts (*Schistocerca gregaria*) consume phytosterols through a plant-based diet, and amplify and metabolize them into new derivatives with potential health benefits for humans. Phytosterols constitute a fraction of unsaponifiable lipids and are encountered either in free form or as fatty acid esters. These compounds lower blood cholesterol levels by inhibiting cholesterol absorption, modulate endothelial function, and exhibit antioxidant, anti-inflammatory, anti-cancer and immune system regulatory effects [31]. In addition, tocopherols have also been detected in some edible insects [33,34]. Hence, there is a need for further research focusing on the content of bioactive compounds (i.e., phenolic compounds, tocopherols and phytosterols) in edible insects, which would demonstrate added value over their already proven beneficial nutritional value. Based on the above observations, we adopted the research hypothesis that the addition of edible insects to nut-based bars will significantly increase their antioxidant potential and the content of biologically active substances. Therefore, the first aim of the study was to determine bioactive compounds from the group of phenolic compounds by means of spectrophotometric and chromatographic methods, to examine the content of tocopherols and phytosterols, and to determine the in vitro antioxidant potential of edible insect flours. The main goal was to determine the effect of the addition of various edible insect flours on the content of bioactive compounds and the antioxidant potential of nut-based bars obtained under laboratory conditions. In addition, we aimed to assess, using sensory evaluation, whether changes in the contents of selected bioactive compounds affect the taste, smell and texture of the modified products.

## 2. Results and Discussion

### 2.1. Polyphenol Profile of Edible Insects and Bars

As already mentioned, edible insects are a valuable alternative source not only of vitamins, minerals, proteins, fats, and calories, but also bioactive compounds from the polyphenol group, as proven in just a few publications in the last few years [26,27,28]. Regarding the analyzed nut bars with the addition of flours from edible insects, the chemical composition (including protein content, amino acid composition, fatty acid profile, etc.) was described in an earlier publication [15]. The abovementioned data are available in Supplementary Materials (Tables S1–S3).

In the quantitative and qualitative profiles of polyphenols obtained with UHPLC-DAD-ESI-MS/MS, protocatechuic aldehyde, ellagic acid, and gallic acid (Table 1) were found to be present in the highest amounts in edible insects among hydroxybenzoic acid derivatives. In buffalo worm flour, the highest content of the abovementioned phenolic acids was noted, because in addition to these, we also detected syringic acid and protocatechuic acid, which were not found in the remaining insect samples. Hydroxycinnamic acids (caffeic, ferulic, *p*-coumaric, chlorogenic and sinapic acids) were detected in each of the edible insects, but cricket flour had the highest amount of these acids—as much as 14 times more ferulic acid than the others (Table 1). The flavonoid content was low in the edible insects tested. There were mainly quercetin-derived flavonols, and cricket flour (Table 1) had the highest amount of these compounds.

In the study of Nino et al. [27], compounds such as gallic, syringic ferulic, *p*-coumaric, chlorogenic and sinapic acids, in addition to quinic and 4-hydroxybenzoic acid, were found in the cricket. On the other hand, the content of quercetin derivatives was not determined in the study by Nino et al. (2021). The quantitative and qualitative profiles of polyphenols in edible insects strictly depend on their diet; hence the profiles of polyphenols and phenolic compounds, even in the same species of edible insects, can differ significantly [26].

Regarding the analyzed bars, it should be noted that the standard bar, which includes honey and nuts, was characterized by a large amount of hydroxybenzoic acids, because the abovementioned ingredients are a source of such acids as gallic, protocatechuic, syringic, ellagic, ferulic, vanillic and chlorogenic, as well as the flavonoids apigenin, kaempferol, quercetin and its derivatives [35,36,37]. The amount of gallic acid, similarly to ellagic acid and 2,5-dihydrobenzoic acid, was higher in the bars with the addition of edible insects compared to the standard bar. Most likely, it is introduced together with the addition of edible insects, because the highest amount of gallic acid was found in mealworm flour bars (4.63 mg/100 g) (Table 1). Among all the ingredients, mealworm flour contained the most of this acid. Similarly, the amount of 2,5-dihydroxybenzoic acid in cricket bars increased, because cricket flour contained the highest amount of this ingredient (0.69 mg/100 g). Ellagic acid was also the most abundant in cricket bars for the same reason. Cricket flour contained the highest amount of this phenolic acid (6.47 mg/100 g) (Table 1). The standard bar was rich in protocatechuic acid, and its amount increased when buffalo worm flour, which was characterized by a large amount of this phenolic acid, was added (Table 2).

### 2.2. Tocopherols and Sterols in Insect Flours and Bars

In this study, α-, δ-, β- and γ-tocopherols were identified in the studied edible insect flours. The presence of tocopherols has been previously reported in other edible insects, such as the larvae of the red palm weevil *Rhynchophorus ferrugineus* and silkworm *Bombyx mori* [34]. A significant difference (*p* < 0.05) in the total amounts of these four tocopherols was observed for the different evaluated edible insect flours (44.20 to 241.05 mg/100 g fat) (Table 3). Of all the analyzed edible insect flours, cricket flour exhibited the highest content of these compounds, while the mealworm flour had the lowest tocopherol level. The predominant tocopherol isomer in cricket flour was β-tocopherol (98.15 mg/100 g fat), followed by γ, α and δ isomers (75.32, 39.81, and 27.78 mg/100 g fat, respectively), while in the case of buffalo worms and mealworm flours, γ-tocopherol was present in the highest amounts (27.91 and 16.59 mg/100 g fat, respectively). In addition, α-tocopherol was also found in slightly smaller amounts in flours from these insects (26.43 and 14.54 mg/100 g fat, respectively).

**Figure 1:**
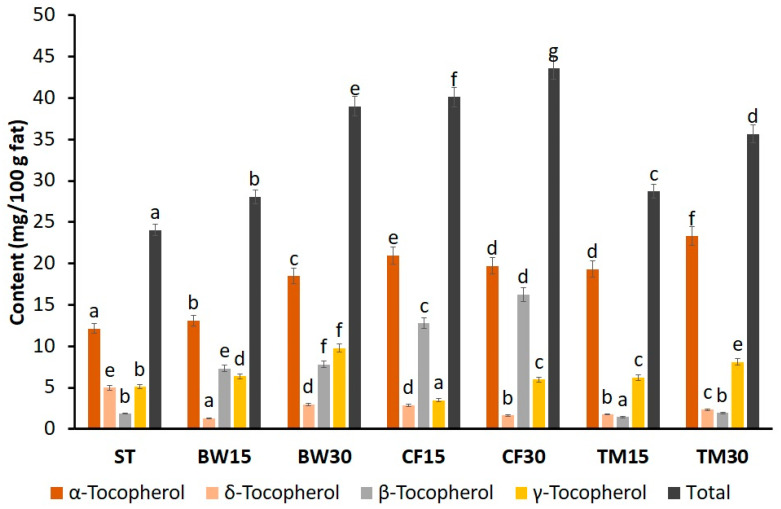
Composition of tocopherols in the standard and edible insect flour-substituted bars (Values marked with the same letter do not differ statistically significantly at *p* < 0.05).

Figure 1 shows the composition of tocopherols in the standard and edible insect flour-substituted bars. It was observed that the total tocopherol content increased from 17 to 81% in bars enriched with insect flour compared to the standard bar. The concentration of tocopherols in the bars was shown to increase in accordance with the additive used. The highest amount of tocopherols was found in the cricket bars (43.57 mg/100 g fat), while the lowest content of these compounds was found in standard bars (24.06 mg/100 g fat). These results are not surprising, as flour from these insects had the highest tocopherol content (Table 3). Considering the profile of individual tocopherols, it was observed that the predominant tocopherol in the bars analyzed was α-tocopherol. Its concentration ranged from 12.12 mg/100 g fat in the standard bar to 32.31 mg/100 g fat in bars with mealworm flour. Nevertheless, the bars with mealworm flour had the lowest δ- and β-tocopherol contents. The contents of these two isomers were even lower than in the standard bar. Interestingly, regardless of the additive used, bars enriched with insect flour showed a significantly lower δ-tocopherol content compared to the standard bar.

The individual and total sterol contents in the studied edible insect flours are presented in Table 3. In the all analyzed edible insect flours, the sterols detected and quantified were cholesterol, campesterol, stigmasterol, clerosterol and β-sitosterol. The total amount of sterols in the analyzed insect flours varied significantly from 516.07 to 1528.04 mg/100 g fat. Cholesterol contributed the most to the total sterol levels (86–88%) in all flours. The levels of cholesterol varied widely among all analyzed fractions of insect flours, from 452.17 mg/100 g of fat in mealworm flour to 1310.41 mg/100 g of fat in cricket flour. Taking into account the fact that the authors often present the cholesterol content in relation to the whole product and not to the content in the fat fraction, these values should be considered as average and characteristic for products of animal origin [38,39]. Other sterols were detected at much lower levels. The content of β-sitosterol, the second most abundant sterol in the insect flours, was significantly lower than the levels of cholesterol, and ranged from 27.67 mg/100 g in mealworm flour to 60.04 mg/100 g fat in cricket flour. The levels of campesterol, clerosterol, and stigmasterol also varied significantly among different types of insects and were within the ranges 12.32–55.60, 7.90–56.68 and 2.80–45.31 mg/100 g fat, respectively. Other authors have also shown that edible insects are a source of phytosterols, and their composition depends mainly on the diet of the insects [31,32]. For example, Ochieng et al. (2022) found that the longhorned grasshopper (*Ruspolia differens* Serville) contains considerable amounts of (22Z)-27-norergosta-5,22-dien-3β-ol, cholesterol, campesterol, cholest-4-ene-3-one and β-sitosterol.

**Figure 2:**
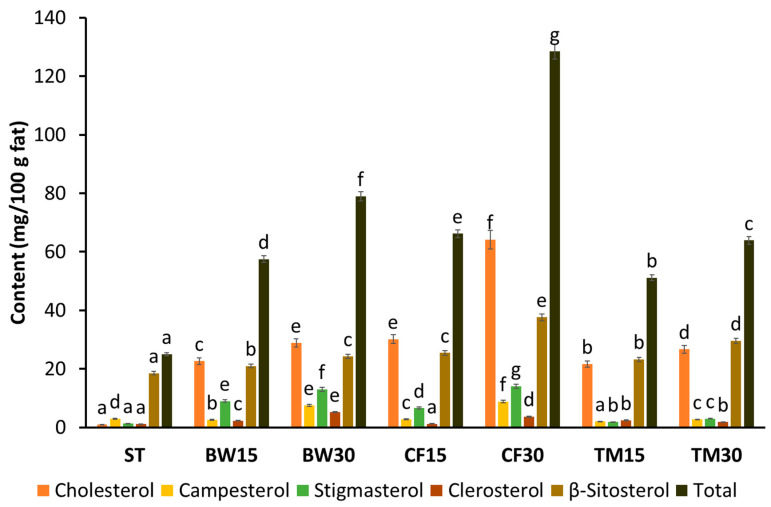
Composition of sterols in the standard and edible insect flour-substituted bars (Values marked with the same letter do not differ statistically significantly at *p* < 0.05).

The results show that the enrichment of bars with insect flour significantly affected the composition of sterols of the studied samples (Figure 2). The total sterol content of the standard bars was found to be similar to that of bars enriched with insect flours, but the concentrations of individual sterols are markedly different. The levels of sterols in the bars depend on the type and share of insect flours used. It was observed that the enrichment of bars with insect flour, regardless of its type, significantly increases the content of sterols, including phytosterols, in bars. The total amount of these compounds varied widely among all tested bars, and ranged from 24.97 to 128.38 mg/100 g fat. Among the bars with insect flour, those enriched with cricket flour at a concentration of 30% had the highest sterol content, while those with a 15% addition of mealworm flour had the lowest contents of these compounds. 

As can be seen in Figure 2, for standard bars, β-sitosterol was the dominant sterol, followed by campesterol. The other sterols—stigmasterol, clerosterol and cholesterol—were present only in small amounts. The most prevalent sterol in the enriched bars was cholesterol, which represented about 13–50% of the total sterol levels. The highest amount of this compound was found in the cricket bars (64.16 mg/100 g fat), while the lowest was found in mealworm bars (21.62 mg/100 g fat). The second most abundant sterol in the edible insect flour-substituted bars evaluated was β-sitosterol (20.97–37.65 mg/100 g fat), followed by stigmasterol (1.84–14.05 mg/100 g fat), campesterol (2.01–8.84 mg/100 g fat), and clerosterol (1.24–5.23 mg/100 g fat). As with cholesterol, bars enriched with cricket flour at a concentration of 30% showed the highest concentration of almost all sterols. The use of other insect flours as an additive, particularly at the lower of the concentrations used, contributed to a slight increase in the concentration of β-sitosterol, campesterol and clerosterol. Interestingly, the enrichment of the bars with buffalo worm and cricket flours contributed to a significant increase in stigmasterol concentrations compared to standard bars. Based on the results, it can be concluded that the enrichment of bars with insect flours, although raising the cholesterol level, makes it possible to increase the level of valuable phytosterols in the final product. Phytosterols can reduce cholesterol absorption and serum LDL cholesterol levels [40]. In addition, the consumption of phytosterols can also increase the activity of antioxidant enzymes and consequently reduce oxidative stress. Beneficial effects caused by the consumption of phytosterols with everyday diet are apparent at doses of approximately 2000 mg/day [40,41].

### 2.3. Antioxidant Potential of Edible Insects and Bars

The total content of polyphenols determined by the spectrophotometric method using the Folin–Ciocalteu reagent is a useful value for correlating the contents of phenolic compounds with their antiradical and antioxidant activities. Therefore, in addition to the determination of the quantitative and qualitative profiles of polyphenols by the chromatographic method (discussed in Section 2.1), the total content of polyphenols was also determined using the semi-quantitative method, although the results obtained with this method, according to some authors [42,43], may be subject to error due to the possibility of the reaction of the Folin–Ciocalteu reagent not only with polyphenols, but also with other compounds, e.g., vitamin C, some alkaloids, amino acids, proteins and polysaccharides, as well as organic acids.

The total amounts of polyphenols in the edible insect flour were as follows: 273.15; 283.26 and 292.37 mg GEA/100 g for mealworm (*Tenebrio molitor*), buffalo worm (*Alphitobius diaperinus*) and cricket (*Acheta domesticus*), respectively (Table 4). In the studies by del Hierro et al. [44], the polyphenol content in *A. domesticus* was 5.0 g GAE/100 g, while a value of approximately 3.8 g GAE/100 g was obtained for *T. molitor*. Musundire et al. [45] reported a value of 3.6 g GAE/100 g for unprocessed edible stinkbugs (*Encosternum delegorguei*). In a similar study, the total phenolic content of unprocessed edible beetle (*Eulepida mashona*) was found to be 0.08 g GAE/100 g of the sample [46]. In another study, the total phenolic content of the edible ground cricket (*Henicus whellani*) was 0.77 g GAE/100 g [47]. Additionally, Liu et al. [48] identified 5.0 g GAE/100 g of sample of *H. parallela*. On the other hand, in the study of Nino et al. [27], TPC was present at only 1.9 g GAE/100 g of the sample.

It should be emphasized that the estimated content of total polyphenols depends on the applied assay method, including the type of extraction, and could be expressed in various ways (e.g., various phenolic acids used to calculate polyphenols) [49]. In the abovementioned studies, various extraction methods were used, including ultrasound-assisted extraction (UAE) and pressurized liquid extraction (PLE), in the studies by del Hierro et al. [44], hence the discrepancies between the research results. Moreover, the edible insect extracts could contain vitamins and free amino acids, which are easily extracted by ethanol and can show reactivity with the Folin–Ciocalteu reagent. As previously mentioned, the Folin–Ciocalteu assay is a well-known method to quantify phenolic compounds in diverse samples. In fact, the reaction mechanism is not specific for phenolics. For example, other reducing compounds, such as ascorbic acid and some amino acids, could contribute to the total phenolic content, leading to an over-estimation of the phenolic compounds in the samples [50].

It should also be added that the content of phenols in insects is closely correlated with their diet; hence, according to Nino et al., there may be discrepancies in total phenolic content for the above samples [27].

It should be emphasized that polyphenols act as antioxidants in several directions: as reducing substances, deactivating free radicals, chelating metals catalyzing oxidation processes, etc. [51,52]. In food, all the abovementioned directions of action of these compounds can be found simultaneously. It can be concluded that the estimation of the antioxidant potential of a product requires the use of many methods of estimating the antioxidant and antiradical activity, because each of them is based on different reaction mechanisms, taking into account the abovementioned multidirectionality of polyphenols as antioxidants. It seems, therefore, that it is justified to analyze the antioxidant and antiradical activities using several methods that will largely define the antioxidant potential of both edible insects and products containing them.

Therefore, to assess the antioxidant capacity of the products examined in this study, we employed techniques that utilize the capability of antioxidants to neutralize stable free radicals (ABTS, DPPH) and to decrease the oxidation state of metal ions through the antioxidant being tested (FRAP) [53]. These approaches are complementary, as the FRAP method encompasses the majority of antioxidant components present in the sample, while the DPPH method only identifies a subset of the most reactive ones, and the ABTS method produces intermediate results [53].

We also used an additional ferric reduction method [54] to estimate the antioxidant activity of primarily newly formed compounds, namely, melanoids, which are a product of the Maillard reaction. Melanoids have antioxidant properties, and also antimicrobial, antihypertensive, antiallergenic, and prebiotic properties. Melanoidins also demonstrate the ability to bind metal ions such as Fe^2+^ (oxidation reaction catalysts) [55], and are considered as antimutagenic and tumor growth-inhibiting compounds [56].

When considering the antioxidant and antiradical activities of edible insects, it was found that cricket had the highest antioxidant potential, regardless of the method of activity determination (Table 4). In this study, the antioxidant activity (using the ABTS method) for *A. domesticus* and buffalo worms was 7.72 and 7.55 mM Tx/100 g, respectively, and FRAP was 7.15 and 6.93 mmol Fe/100 g (Table 4). In the study by Di Mattia et al. [57], *A. domesticus* showed antioxidant activity (using the ABTS method) of about 2.37 mM Tx/100 g, and buffalo worms 0.82 mM Tx/100 g. In the case of the FRAP method, *A. domesticus* showed activity at the level of 1.81 mmol Fe/100 g. According to Mattia et al. (2019), the antioxidant activity of edible insects can be 2–3 times higher than orange juice or olive oil, although they contain less TPC. The correlations between TPC and DPPH, ABTS and FRAP were on an average level, at 0.659, 0.4490 and 0.4760, respectively. On the other hand, the correlation between TPC and the ferric reducing method was negative (−0.1172). Therefore, we can infer that the antiradical and antioxidant activity not only depended on polyphenols, but also on other antioxidant compounds. These compounds include tocopherols, and especially proteins [58,59]. In this study, cricket flour had the highest content of tocopherols, and therefore had the highest antioxidant potential (Table 3 and Table 4). The results of Di Mattia [57] show that the antioxidant pattern of the edible insects and invertebrates is different according to taxonomy and dietary habits.

Considering the effect of the addition of edible insects to the bars in the amounts of 15 and 30%, the total content of polyphenols increased from 12 to 62% compared to the control bar (Table 5). This bar consisted of honey, hazelnuts and cashew nuts, which also contain a very large amount of polyphenols. It was found that the amount of polyphenols in the bars increases proportionally to the additive used, and the highest amount of polyphenols was found in the cricket bars, because there were large quantities of polyphenols in the cricket flour itself (Table 4 and Table 5).

Compared to the standard bar, it was found that the antiradical activity of the edible insect bars was higher in the range of 36–82% (DPPH method) and in the range of 23–87% (ABTS method). In the case of antioxidant activity determined by the FRAP method, the bars with edible insects showed 33 to 81% higher antioxidant activity compared to the standard (Table 5). It was shown that each 30% addition of edible insects to the bars guaranteed greater antiradical and antioxidant activity in each of the three methods than a 15% addition of these insects. Among all the analyzed bars with edible insects, the highest antioxidant potential was shown by bars with 30% buffalo worm flour, regardless of the method of determination (Table 5).

Such high antiradical and antioxidant activity of the abovementioned bar with 30% buffalo worm flour can be explained by the fact that the antiradical and antioxidant properties of the sample result not only from TPC components, but above all from the quantitative and qualitative profiles of these compounds. Considering the fact that medium correlations were found between TPC and DPPH, ABTS and FRAP (0.7067, 0.6466, and 0.7075 respectively) and between TPC and the ferric reduction method (−0.6642), it should be concluded that the high antioxidant potential of bars with 30% buffalo worm flour depends on the amount of individual phenolic compounds in this sample. It should also be noted that phenolic compounds show different activities in relation to different free radicals and methods of determining their activity [60]. According to many authors [60,61,62], phenolic acids such as gallic, caffeic, syringic, sinapic, ferulic, and protocatechuic acids show the highest efficiency of scavenging DPPH and ABTS radicals. Moreover, according to Gadow et al. [63], the power of deactivation of DPPH and ABTS radicals shown by flavonols is comparable to the abovementioned phenolic acids. In the case of the FRAP method, the following acids should be taken into account in particular: gallic, caffeic and ferulic [60,62]. The highest activity of the abovementioned sample resulted in this case from the largest amounts of effective phenolic acids, such as syringic, protocatechuic, protocatechuic aldehyde and caffeic acid, and significant amounts of gallic acid and quercetin derivatives, among all bars with edible insect flour. 

In addition, other substances could contribute to the antioxidant capacity, e.g., high-molecular Maillard reaction products formed at high temperatures (during roasting nuts), which also have antioxidant properties. The samples in which the most melanoids were formed were the bars with 30% buffalo worm flour, with the lowest ferric reduction EC_50_ value (46.08 mg/mL). Additionally, other components, e.g., carotenoids, glutathione, and inositol phytates, the contents of which were not determined in this work [64,65,66], can influence antioxidant activity. Tocopherols and phytosterols also show antioxidant activity. Taking into account the fact that the in vitro antioxidant activity of tocopherols is in the series alpha < beta < gamma < delta [67], it can be noted that among the bars with edible insects discussed above, the highest amount of gamma tocopherol was found in the sample bars with 30% buffalo worm flour (Figure 1), which additionally contributed to the greatest antioxidant potential of this sample (Table 5).

### 2.4. Sensory Analysis

Nutritional value must be accompanied by sensory appeal, as consumer acceptance is one of the key parameters for determining the marketability of a new product. Smell, taste and texture are the most important factors that determine the consumer’s purchasing decision. 

**Figure 3:**
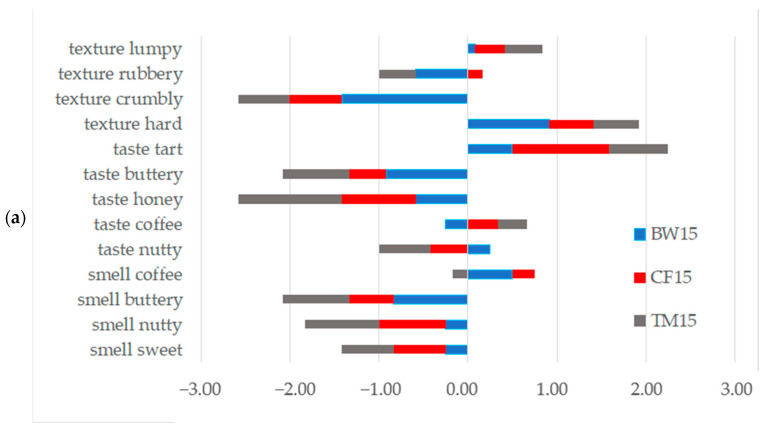

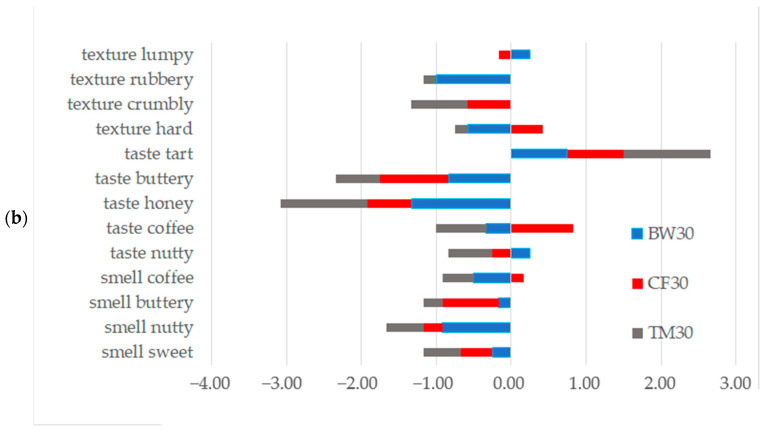
Directions of changes in features assessed in sensory analysis: (**a**) bars with 15% of insect flours; (**b**) bars with 30% of insect flours.

The use of insect flour as an additive resulted in slight changes in perceived smell perception (−0.92 to 0.50) and some changes in texture (−1.42 to 0.92) of peanut bars. The greatest impact was observed in perceived taste perception (from −1.33 to 1.17). The additives used (regardless of their size) reduced the intensity of perceived sensations relating to the sweet, nutty, buttery and honey flavors, compared to the standard bar. A higher intensity of the perceived sensation was noted only for the tart taste. For the other descriptors, the directions of changes varied and depended on the amount of addition and the type of insect (Figure 3).

The results obtained seem to be confirmed by data from the literature. For example Ruszkowska et al. [14] observed that, in extruded corn snacks with cricket flour, a greater than 6% addition of cricket flour led to negative changes in the texture of the resulting snacks, as reflected in the lower consumer acceptability [14]. Pauter et al. [11] observed in muffins with cricket flour an increase in acceptance in taste and texture ratings compared to muffins without insect flour, although some consumers indicated a perceptible “unpleasant” taste. The authors predict that a share of 2% would be acceptable to consumers [11]. Kowalski et al. [5], in breads supplemented with buffalo worm, cricket, and mealworm powder, found that the presence of a 10% share of these flours in breads did not lower consumer acceptance [5]. In another study regarding bars containing insect flours, a reduction in consumer evaluation of the products was observed. Reduction in consumer acceptance was mostly visible in the case of cricket flour addition [15]. On the other hand, a significant effect of mealworm flour on appearance, smell, taste and texture of the final product was observed in the case of sponge cake supplementation with the abovementioned additive [10]. Changes in the smell profile may result from the amino acid composition of insect proteins, and thus the presence of precursors of volatile compounds shaping sensory sensations [9].

## 3. Materials and Methods

### 3.1. Materials

Nut bars were obtained under laboratory conditions using hazelnuts (Bakalland Ltd., Warsaw, Poland) and cashews (Bakalland Ltd., Warsaw, Poland) in a 1:1 ratio and honeydew honey (Huzar Ltd., Nowy Sacz, Poland) as a binder. The amount of honey was 0.75% based on the weight of the nuts. In the bars with edible insect flour, the addition of 15 and 30% insect flour from mealworm *Tenebrio molitor* (TM) (DeliBugs, Lelystad, Netherlands), buffalo worm *Alphitobius diaperinus* (BW) (DeliBugs, Lelystad, Netherlands), or cricket *Acheta domesticus* (CF) (DeliBugs, Lelystad, Netherlands), based on the weight of the nuts, was applied.

#### Preparation of Nut Bars with Insect Powder

A mix of nuts was roasted at 180 °C for 5 min using a pan. The crushed nuts were mixed with dissolved honey (35 °C) and transferred to silicone molds (75 mm × 30 mm × 30 mm) and kept at 4 °C for 120 min.

### 3.2. Methods

Ethanol extracts used for the determination of antioxidant constituents were obtained by dissolving 0.6 g of the sample in 30 mL of 80 g/100 g ethanol for 120 min (electric shaker: type WB22, Memmert, Schwabach, Germany). The obtained extracts were centrifuged (15 min, 4500 rpm 1050× *g*) in a centrifuge type MPW-350 (MPW MED, Instruments, Warsaw, Poland). The supernatant was decanted and stored at −20 °C for further analyses. 

#### 3.2.1. Phenolic Compounds Analysis by UHPLC-DAD-ESI-MS/MS

The study analyzed phenolic compounds in extracts obtained from edible insect flour and bars. The method used for the analysis was UHPLC-DAD-ESI-MS/MS as described by Oracz et al. (2019). The UHPLC-DAD analysis was conducted using a UHPLC+ Dionex UltiMate 3000 liquid chromatographic system equipped with a diode array detector with multiple wavelengths (Thermo Fisher Scientific Inc., Waltham, MA, USA), while the UHPLC-ESI-MS/MS analysis was performed on a Transcend TLX-2 multiplexed LC system equipped with a Q-Exactive Orbitrap mass spectrometer (Thermo Scientific, Hudson, NH, USA) using a heated electrospray ionization interface (HESI–II). The separation of phenolic compounds was done using an Accucore C18 column (2.1 × 150 mm, 2.6 μm particle size) kept at 30 °C. The mobile phase and gradient program were used as previously described by Oracz et al. (2019), with some modifications. The mobile phases were eluent A, 0.1% formic acid in water (*v*/*v*), and eluent B, acetonitrile. The flow rate was 0.35 mL/min, and the gradient was as follows: 0–8 min, 1–5% B; 8–15 min, 5–8% B; 15–20 min, 8–10% B; 20–25 min, 10–15% B; 25–35 min, 15–20% B; 35–40 min, 20–25% B; 40–50 min, 25–90% B; 50–53 min, 90% B; 53–58 min, 90–1% B; finally, the initial conditions were held for 7 min for column re-equilibration. The chromatograms were recorded at two different wavelengths (i.e., 280 nm for hydroxybenzoic acids and their derivatives and 320 nm for hydroxycinnamic acids and their derivatives). The mass spectrometry conditions were set as follows: the capillary voltage was 4500 V, the capillary temperature was maintained at 275 °C, the heater gas was set to 320 °C, and nitrogen was used as both the sheath and auxiliary gas with flow rates of 35 and 15 (arbitrary units), respectively. The mass spectrometer acquired full scan mass spectra in negative ion mode over a range of *m*/*z* 100 to 1500. MS/MS spectra were obtained using collision-induced dissociation (CID) mode with a normalized collision energy (NCE) of 20%. To identify the phenolic compounds, their retention times, UV-vis absorbance spectra, full scan mass spectra, and MS/MS fragmentation patterns were compared with those of corresponding standards analyzed under the same conditions and reports from previous studies [68]. 

The quantification of each phenolic compound was performed using the external standard method. To create calibration curves, external standard solutions were injected in triplicate at concentrations ranging from 0.01 to 100 mg/L. The linearity range was determined based on the regression curves and correlation coefficients. The limit of detection (LOD) and limit of quantification (LOQ) were calculated as the minimum concentrations that yielded a signal-to-noise ratio of at least 3-fold (S/N ≥ 3) and 10-fold (S/N ≥ 10), respectively. In this work, gallic acid, protocatechuic acid, ellagic acid, vanillic acid, *p*-hydroxybenzoic acid, syringic acid, caffeic acid, ferulic acid, *p*-coumaric acid, chlorogenic acid, sinapic acid, 3,4-di-O-caffeoylquinic acid, 2,5-dihydroxybenzoic acid, quercetin 3-*O*-galactoside, quercetin 3-*O*-glucoside and quercetin 3-*O*-rutinoside (rutin) were quantified using the corresponding reference standards. The quantity of protocatechuic aldehyde was calculated on the basis of the protocatechuic acid standard curve. The phenolic compound content was expressed as mg per 100 g of sample (mg/100 g).

#### 3.2.2. Determination of Tocopherols and Sterols

The tocopherol and sterol profiles were determined by GC with FID (GC/FID) after saponification with 2 M methanolic KOH solution followed by extraction with n-hexane [69]. Samples were analyzed using a Shimadzu GC2010Plus Chromatograph (Shimadzu corp., Kyoto, Japan) equipped with an FID detector. The separation of tocopherols and sterols was carried out using an SH-5MS (30 m × 0.25 mm × 0.25 μm) chromatograph (Shimadzu corp., Kyoto, Japan). The injector temperature was set to 280 °C, and a temperature program was implemented, including isothermal holding at 285 °C for 5 min, followed by an increase to 290 °C at a rate of 5 °C/min and isothermal holding for 19 min. The carrier gas utilized was nitrogen, flowing at a rate of 0.92 mL/min with a split ratio of 1:10. Phytosterols were identified by comparing their retention times and mass spectra with those of standards and data from the literature [70,71]. The measurements were carried out three times, and the results are reported in milligrams per 100 g of fat.

#### 3.2.3. Analysis of Polyphenols, Flavonoids and Antioxidant Potential

- Total content of polyphenols

The Folin–Ciocalteu reagent method, as described by Singleton et al. [72], was used to measure the total polyphenol content. In brief, 5 mL of the extract was diluted with distilled water to make a final volume of 50 mL. To 5 mL of the diluted extract, 0.25 mL of Folin–Ciocalteu reagent (previously diluted with distilled water in a 1:1 *v*/*v* ratio) and 0.5 mL of 7% Na_2_CO_3_ were added. The mixture was then vortexed using a WF2 vortex mixer (Janke & Kunkel, Staufen, Germany) and stored in a dark place for 30 min. The absorbance was measured at 760 nm using a Helios Gamma 100–240 spectrophotometer (Runcorn, UK). The results are reported as milligrams of catechin equivalent per 100 g of dry mass of the sample (mg catechin/100 g D.M.).

- Determination of flavonoids

The flavonoid content was determined using a spectrophotometric method described by El Hariri et al. [73]. In summary, 0.5 mL of the extract was mixed with 1.8 mL of distilled water and 0.2 mL of 2-aminoethyldiphenylborate reagent in a test tube. The mixture was vortexed, and the absorbance was measured at a wavelength of 404 nm. The flavonoid content was expressed in milligrams of rutin equivalent per 100 g of dry mass of the sample (mg RE/100 g D.M.).

- Free radical scavenging activity by DPPH

The free radical scavenging activity of samples was measured using the 2,2-diphenyl-1-picrylhydrazyl (DPPH) by the method of Sánchéz-Moreno et al. [74]. The extract (0.4 mL) was mixed with 3.6 mL of DPPH solution (0.025 g DPPH in 100 mL of methanol). The absorbance of the reaction mixture was determined using a Jenway spectrophotometer (6405 UV/Vis, England) at 515 nm. Trolox (6-hydroxy-2,5,7,8-tetramethylchroman-2-carboxylic acid) (10–100 mg/L; R2 = 0.989) was used as the standard, and the results are expressed in mg/g Trolox equivalents.

- Antiradical activity by ABTS

Antiradical activity was assessed using the synthetic radical cation ABTS according to Re et al. [75]. The radical scavenging activity was measured using an ABTS (2,2, azino-nis (3-ethylbenzthiazoline-6-sulfuric acid)) radical cation decoloration assay. The bleaching rate of ABTS^+•^ in the presence of the sample was monitored at 734 nm using a Helios Gamma 100–240 (Runcorn, UK) spectrophotometer. The ABTS^+•^ stock solution was diluted in PBS buffer up to an Abs of 0.700 ± 0.05 for the analysis of extracts. Volumes of 2.00 mL of ABTS^+•^ and respective dilution extracts into PBS buffer solution were used. The ABTS^+•^ bleaching was monitored at 30 °C and the decoloration after 6 min was used as the measure of antiradical activity. Radical scavenging activity was measured as Trolox equivalents antioxidant capacity (mg Tx/g of sample).

- Ferric reducing antioxidant power (FRAP)

The Oyaizu method [76] was used to evaluate the reducing power of the samples. Initially, 1 mL of the sample extract was mixed with 5 mL of PBS (phosphate buffer with pH 6.6) and 5 mL of 1% potassium ferricyanide in a test tube. The resulting mixture was then stirred and heated in a water bath at 50 °C for 20 min. After cooling, 5 mL of 10% trichloroacetic acid was added, followed by transferring 5 mL of the resulting solution into a new test tube. Then, 5 mL of distilled water and 1 mL of 0.1% ferric chloride solution were added, and the absorbance at 700 nm was measured using a Jenway spectrophotometer (6405 UV/VIS, England). The results were expressed in mg/g Trolox equivalents, and Trolox (10–100 mg/L; R2 = 0.9974) was used as the standard for comparison.

- Determination of ferrous ion chelating activity

The chelation of Fe(II) ions by the ethanol extracts was determined as described by Oracz and Żyżelewicz (2019). Briefly, 1 mL of each extract or blank was mixed with 1.85 mL of high-purity deionized water and 50 µL of 2.0 mM FeCl_2_. The solution was mixed for 30 s, and then 100 µL of 5 mM ferrozine was added. The reaction mixture was vortexed and left to stand at room temperature for 15 min. The absorbance of the solution was measured spectrophotometrically at 562 nm with a Helios Gamma 100–240 (Runcorn, UK). A calibration curve was constructed using disodium ethylenediaminetetraacetate dihydrate (EDTA). All experiments were carried out in triplicate and the results are expressed as mg EDTA/g dm [54].

#### 3.2.4. Sensory Analysis—Quantitative Descriptive Analysis

Sensory analysis was based on the 13299:2016 standard [77]. The sensory analysis of the prepared bars was carried out by a team of 12 experts. In the first stage, the evaluators familiarized themselves with the product and, under the guidance of the leader, selected descriptors appropriate for the product, as well as a rating scale. Then they verified the prepared list by testing several bars. The proper assessment included a set of 7 samples (6 with the addition of insects and a reference sample, the so-called standard), presented in a random order. Selected characteristics were assessed as a deviation from those present in the reference product, i.e., bars without the addition of insects. A bipolar scale (10 cm long) with a marked standard in the middle of the scale was used for the assessment. Deviation to the left meant the intensity of the discriminant was lower than in the reference product, and deviation to the right meant it was higher. The team assessed the smell (sweet, nutty, buttery, coffee), the taste (nutty, coffee, honey, buttery, tart) and the texture (hard, crumbly, rubbery, lumpy) of the presented products. The obtained results are presented in the form of differential profilograms. All participants voluntarily signed consent forms for participation in the study, which was approved by the Independent Bioethics Committee for Research of the Medical University of Gdańsk (NKBBN/346/2021). This study is in line with the ethical principles of non-violence, beneficence, justice and autonomy contained in the ethical provisions of the 2013 Revised Declaration of Helsinki. 

## 4. Statistical Analysis

The results are expressed as the arithmetic mean ± the standard deviation. Significance of differences was demonstrated by analysis of variance at a significance level of *p* ≤ 0.05 and Fisher’s post-hoc test. Calculations were made using the Statistica 13.0 (StatSoft, Kraków, Poland) software.

## 5. Conclusions

The inclusion of edible insects in the human diet can make them a suitable, healthy, and sustainable alternative to traditional animal products. This is possible not only because of their content of essential nutrients, such as good-quality biological protein or omega-3 and omega-6 fats, but also antioxidant components. In addition, the form of consumption and the wide range of products to which insects or their preparations can be added offer a number of advantages. This provides an opportunity to introduce valuable proteins into the diets of those who, for various reasons, do not accept the consumption of animal flesh. Research into the use of edible insects in snacks, pastries, bakery products, etc., offers the possibility of gradually promoting and implementing edible insects in human nutrition. Based on the research conducted, it can be surmised that edible crickets and their preparations may have the greatest nutritional potential, and therefore it is worth undertaking further research into their safe use in human nutrition.

The analysis of the obtained products shows that the incorporation of insect flours into nut bars contributed to an increase in their antiradical and antioxidant potential. Among all tested samples, the highest antioxidant potential (14.74 ± 0.23 mg Tx/g) was found in bars with 30% buffalo worm flour. This addition also contributed to their enrichment in valuable hydroxybenzoic acids and hydroxycinnamic acids (especially protocatechuic, syringic, and chlorogenic acid in the amounts of 53.10, 18.00, and 3.28 mg/100 g, respectively). The addition of edible insect powder resulted in a significant increase in the tocopherol content compared to the standard bar, and the dominant tocopherol in the bars was α-tocopherol. While the addition of insect flour contributed to the increase in the cholesterol level, enrichment with insect flour allows the level of valuable phytosterols in the final product to be increased. In addition, bars enriched with cricket flour at a concentration of 30% showed the highest amount of almost all sterols. The ability of phytosterols to reduce cholesterol absorption and serum LDL cholesterol levels, as well as to increase the activity of antioxidant enzymes and thereby reduce oxidative stress, is responsible for the beneficial effects induced by phytosterol consumption. However, it should be borne in mind that the supplementation of products with the use of flours from edible insects contributes to increasing the supply of cholesterol, which may be important for some groups of consumers, for whom increased cholesterol supply is not desirable. The addition of edible insect flours reduced the perception of most sensory attributes of the bars, compared to the standard bar. Especially, in all bars, regardless of the type and percentage of individual edible insects, the sensory panel indicated a more perceptible tart taste of the bars, compared to the standard.

## Figures and Tables

**Table 1:** Profile of phenolic compounds of edible insect flours.

Compounds	Type of Insects		
	Buffalo Worm (*A. diaperinus*)	Cricket (*A. domesticus*)	Mealworm (*T. molitor*)
Hydroxybenzoic acids and their derivatives (mg/100 g)			
Gallic	2.46 ± 0.06 ^b^	0.61 ± 0.02 ^a^	4.63 ± 0.12 ^c^
Vanillic	0.93 ± 0.02 ^b^	0.61 ± 0.02 ^a^	1.36 ± 0.03 ^c^
Protocatechuic	52.14 ± 0.37 ^b^	n.d.	n.d.
Protocatechuic aldehyde	19.81 ± 0.25 ^b^	2.89 ± 0.07 ^a^	23.19 ± 0.26 ^a^
Syringic	26.56 ± 0.44 ^c^	0.46 ± 0.01 ^b^	n.d.
2,5- Dihydroxybenzoic	0.40 ± 0.01 ^b^	0.69 ± 0.02 ^c^	0.15 ± 0.00 ^a^
Ellagic	0.42 ± 0.01 ^b^	6.47 ± 0.16 ^c^	0.26 ± 0.01 ^a^
Hydroxycinnamic acids (mg/100 g)			
Caffeic	0.10 ± 0.01 ^b^	0.11 ± 0.01 ^b^	0.06 ± 0.01 ^a^
Ferulic	0.45 ± 0.04 ^a^	7.23 ± 0.04 ^c^	0.54 ± 0.01 ^b^
*p*-Coumaric	0.08 ± 0.01 ^a^	0.05 ± 0.01 ^a^	0.06 ± 0.01 ^a^
Chlorogenic	0.19 ± 0.02 ^b^	0.20 ± 0.02 ^b^	n.d.
Sinapic	0.34 ± 0.03 ^b^	0.82 ± 0.08 ^c^	0.15 ± 0.01 ^a^
Flavonols (mg/100 g)			
Quercetin 3-O-galactoside	n.d.	0.14 ± 0.01 ^a^	n.d.
Quercetin 3-O-glucoside	n.d.	1.38 ± 0.06 ^b^	0.40 ± 0.01 ^a^
Quercetin 3-O-rutinoside (Rutin)	n.d.	1.38 ± 0.09 ^b^	0.40 ± 0.02 ^a^
Quercetin	n.d.	n.d.	n.d.
Myricetin	n.d.	n.d.	n.d.

Values in the same row within a selected group marked with different letters are statistically significantly different at *p* < 0.05; n.d.—not detected.

**Table 2:** Profile of phenolic compounds of edible insect bars.

Compound	Type of the Bar						
	ST	BW15	BW30	CF15	CF30	TM15	TM30
Hydroxybenzoic acids and their derivatives (mg/100 g)							
Gallic	0.04 ± 0.00 ^a^	0.20 ± 0.00 ^d^	0.37 ± 0.01 ^e^	0.04 ± 0.01 ^a^	0.11 ± 0.00 ^b^	0.16 ± 0.00 ^c^	1.45 ± 0.04 ^f^
Vanillic	0.20 ± 0.03 ^b^	0.11 ± 0.02 ^a^	0.24 ± 0.01 ^b^	0.09 ± 0.00 ^a^	0.22 ± 0.01 ^b^	0.12 ± 0.00 ^a^	0.22 ± 0.01 ^b^
Protocatechuic	41.41 ± 1.4 ^b^	45.10 ± 0.36 ^c^	53.10 ± 0.84 ^d^	40.92 ± 1.03 ^b^	31.96 ± 0.80 ^a^	40.16 ± 1.01 ^b^	33.95 ± 0.85 ^a^
Protocatechuic aldehyde	0.48 ± 0.01 ^a^	1.10 ± 0.03 ^d^	2.20 ± 0.06 ^f^	0.61 ± 0.02 b	0.66 ± 0.02 ^b^	0.94 ± 0.02 ^c^	1.32 ± 0.03 ^e^
Syringic	12.14 ± 0.30 ^c^	14.67 ± 0.31 ^e^	18.00 ± 0.19 ^f^	13.74 ± 0.34 ^d^	13.92 ± 0.35 ^d^	4.51 ± 0.11 ^b^	3.81 ± 0.10 ^a^
2,5- Dihydroxybenzoic	n.d.	0.12 ± 0.01 ^a^	0.37 ± 0.00 ^e^	0.23 ± 0.01 ^c^	0.44 ± 0.01 ^f^	0.18 ± 0.00 ^b^	0.29 ± 0.01 ^d^
Ellagic	0.63 ± 0.02 ^d^	0.40 ± 0.01 ^c^	0.38 ± 0.01 ^bc^	1.15 ± 0.03 ^e^	1.59 ± 0.04 ^f^	0.36 ± 0.01 ^b^	0.31 ± 0.01 ^a^
Hydroxycinnamic acids (mg/100 g)							
Caffeic	1.68 ± 0.07 ^d^	0.08 ± 0.01 ^a^	0.94 ± 0.09 ^c^	0.07 ± 0.01 ^a^	0.41 ± 0.04 ^b^	0.36 ± 0.04 ^b^	0.34 ± 0.03 ^b^
Ferulic	0.18 ± 0.02 ^b^	0.05 ± 0.00 ^a^	0.04 ± 0.00 ^a^	0.28 ± 0.03 ^c^	0.85 ± 0.08 ^d^	0.04 ± 0.00 ^a^	0.04 ± 0.00 ^a^
*p*-Coumaric	n.d.	0.04 ± 0.01 ^a^	0.07 ± 0.01 ^a^	0.06 ± 0.01 ^a^	0.05 ± 0.01 ^a^	0.05 ± 0.01 ^a^	0.07 ± 0.01 ^a^
Chlorogenic	3.45 ± 0.02 ^e^	1.42 ± 0.06 ^b^	3.28 ± 0.03 ^e^	0.58 ± 0.06 ^a^	1.95 ± 0.01 ^d^	1.91 ± 0.02 ^d^	1.62 ± 0.07 ^c^
Sinapic	n.d.	0.20 ± 0.02 ^b^	0.17 ± 0.02 ^b^	0.21 ± 0.02 ^b^	0.18 ± 0.01 ^b^	0.06 ± 0.01 ^a^	0.15 ± 0.02 ^b^
Flavonols (mg/100 g)							
Quercetin 3-O-galactoside	0.09 ± 0.01 ^a^	0.12 ± 0.01 ^a^	0.26 ± 0.03 ^b^	0.09 ± 0.01 ^a^	0.70 ± 0.01 ^c^	0.09 ± 0.01 ^a^	0.08 ± 0.01 ^a^
Quercetin 3-O-glucoside	0.27 ± 0.03 ^c^	0.26 ± 0.03 ^c^	0.25 ± 0.03 ^c^	0.11 ± 0.01 ^a^	0.08 ± 0.01 ^a^	0.29 ± 0.03 ^c^	0.14 ± 0.01 ^b^
Quercetin 3-O-rutinoside (Rutin)	0.71 ± 0.07 ^e^	0.11 ± 0.01 ^a^	0.46 ± 0.05 ^d^	0.16 ± 0.02 ^ab^	0.27 ± 0.01 ^c^	0.76 ± 0.08 ^e^	0.21 ± 0.02 ^b^
Quercetin	n.d.	0.03 ± 0.01 ^a^	0.03 ± 0.00 ^a^	n.d.	0.04 ± 0.00 ^a^	0.03 ± 0.00 ^a^	n.d.
Myricetin	0.06 ± 0.01 ^a^	0.08 ± 0.01 ^a^	0.05 ± 0.01 ^a^	0.06 ± 0.01 ^a^	0.07 ± 0.01 ^a^	0.06 ± 0.01 ^a^	0.05 ± 0.00 ^a^

Values in the same row marked with different letters are statistically significantly different at *p* < 0.05; n.d.—not detected.

**Table 3:** Contents of tocopherols and sterols (mg/100 g fat) in flours from edible insects.

Compounds	Type of Insects		
	Buffalo Worm (*A. diaperinus*)	Cricket (*A. domesticus*)	Mealworm (*T. molitor*)
Tocopherols (mg/100 g fat)			
α-Tocopherol	26.43 ± 1.33 ^b^	39.81 ± 0.33 ^c^	14.54 ± 0.73 ^a^
δ-Tocopherol	5.43 ± 0.27 ^a^	27.78 ± 0.23 ^c^	7.19 ± 0.36 ^b^
β-Tocopherol	7.75 ± 0.39 ^b^	98.15 ± 0.82 ^c^	5.88 ± 0.30 ^a^
γ-Tocopherol	27.91 ± 1.40 ^b^	75.32 ± 0.63 ^c^	16.59 ± 0.83 ^a^
Total	67.52 ± 6.79 ^b^	241.05 ± 4.02 ^c^	44.20 ± 4.45 ^a^
Sterols (mg/100 g fat)			
Cholesterol	535.73 ± 0.77 ^b^	1310.41 ± 0.88 ^c^	452.17 ± 0.65 ^a^
Campesterol	26.38 ± 0.27 ^b^	55.60 ± 0.36 ^c^	12.32 ± 0.10 ^a^
Stigmasterol	2.80 ± 0.05 ^a^	45.31 ± 0.12 ^c^	6.92 ± 0.23 ^b^
Clerosterol	7.90 ± 0.34 ^a^	56.68 ± 0.67 ^c^	16.99 ± 0.45 ^b^
*β*-Sitosterol	40.39 ± 0.47 ^b^	60.04 ± 0.45 ^c^	27.67 ± 0.42 ^a^
Total	613.20 ± 4.34 ^b^	1528.04 ± 4.78 ^c^	516.07 ± 3.22 ^a^

Values in the same row marked with different letters are statistically significantly different at *p* < 0.05.

**Table 4:** Contents of polyphenols and antioxidant activities of flours from edible insects.

		Type of Insects		
	Unit of Measure	Buffalo Worm (*Alphitobius diaperinus*)	Cricket (*Acheta domesticus*)	Mealworm (*Tenebrio molitor*)
Total Phenolic Content	(mg catechin/100 g)	560.55 ± 0.77 ^b^	578.60 ± 1.54 ^c^	539.76 ± 2.32 ^a^
	(mg Gallic acid/100 g)	283.26 ± 0.63 ^b^	292.37 ± 1.37 ^c^	273.15 ± 2.03 ^a^
DPPH	(mg Tx/g)	15.67 ± 0.1 ^a^	16.27 ± 0.1 ^b^	15.85 ± 0.09 ^a^
ABTS	(mg Tx/g)	18.90 ± 0.14 ^a^	19.32 ± 0.14 ^c^	19.12 ± 0.07 ^b^
	(mM Tx/100 g)	7.55 ± 0.08 ^a^	7.72 ± 0.05 ^c^	7.64 ± 0.03 ^b^
FRAP	(mM Fe/kg)	69.3 ± 0.67 ^a^	71.15 ± 0.5 ^b^	70.22 ± 1.48 ^b^
Ferric reduction EC_50_	(mg/mL)	51.28 ± 0.78 ^c^	11.76 ± 0.13 ^a^	18.02 ± 0.12 ^b^

Values in the same row marked with different letters are statistically significantly different at *p* < 0.05.

**Table 5:** Content of polyphenols and antioxidant activity of edible insect bars.

	Unit of Measure	Type of Bar						
		ST	BW15	BW30	CF15	CF30	TM15	TM30
Total phenolic content	(mg catechin/100 g)	190.19 ± 3.09 ^a^	221.92 ± 0.00 ^c^	251.46 ± 1.54 ^f^	241.61 ± 1.54 ^e^	309.45 ± 1.54 ^g^	213.17 ± 6.18 ^b^	238.33 ± 1.54 ^d^
	(mg gallic acid/100 g)	96.38 ± 2.89 ^a^	112.07 ± 0.00 ^c^	127.30 ± 1.38 ^f^	122.22 ± 1.21 ^e^	156.7 ± 1.43 ^g^	108.02 ± 3.01 ^b^	120.70 ± 1.16 ^d^
DPPH	(mg Tx/g)	6.52 ± 0.04 ^a^	9.94 ± 0.03 ^d^	11.87 ± 0.00 ^f^	8.87 ± 0.00 ^b^	10.86 ± 0.14 ^e^	9.32 ± 0.00 ^c^	9.84 ± 0.32 ^d^
ABTS	(mg Tx/g)	7.87 ± 0.02 ^a^	12.01 ± 0.43 ^d^	14.74 ± 0.23 ^e^	9.72 ± 0.10 ^b^	13.10 ± 0.32 ^d^	11.25 ± 0.00 ^c^	11.88 ± 0.42 ^d^
	(mM Tx/100 g)	3.15 ± 0.01 ^a^	4.70 ± 0.03 ^d^	5.82 ± 0.02 ^e^	3.83 ± 0.01 ^b^	5.14 ± 0.02 ^d^	4.44 ± 0.00 ^c^	4.69 ± 0.04 ^d^
FRAP	(mM Fe/kg)	28.67 ± 0.15 ^a^	44.4 ± 0.25 ^d^	51.8 ± 0.14 ^f^	38.2 ± 0.42 ^b^	48.10 ± 0.07 ^e^	40.7 ± 0.27 ^c^	43.5 ± 0.33 ^d^
Ferric reduction EC_50_	(mg/mL)	416.67 ± 1.16 ^g^	151.58 ± 1.25 ^f^	46.08 ± 0.57 ^a^	70.63 ± 1.28 ^d^	49.12 ± 0.19 ^b^	80.56 ± 0.00 ^e^	59.71 ± 0.00 ^c^

Values in the same row marked with different letters are statistically significantly different at *p* < 0.05.

## Data Availability

Not applicable.

## Supplementary Materials

The following supporting information can be downloaded at: https://www.mdpi.com/article/10.3390/molecules28083556/s1.

## Sample Availability

Samples of the compounds are available from the authors.

## Abbreviations

BW—buffalo worm flour, CF—cricket flour, TM—*Tenebrio molitor* flour (mealworm flour), ST—standard bar, BW15 and BW30—bars with 15% and 30% additions of buffalo worm flour, CF15 and CF30—bars with 15% and 30% addition of cricket flour, TM15 and TM30—bars with 15% and 30% addition of *Tenebrio molitor* flour, CN—cashew nuts, HN—hazelnuts, GAE—gallic acid equivalents, TPC—total phenolic content.
