# Supplementary material for: Bioactive Compounds and Antioxidant Composition of Nut Bars with Addition of Various Edible Insect Flours
Source: Molecules. 2023 Apr 18;28(8):3556. doi: 10.3390/molecules28083556 (PMC10143157; doi:10.3390/molecules28083556)
Supplement: Supplementary file 1 [file molecules-28-03556-s001.zip › molecules-2355941-supplementary.pdf]

Table S1. Chemical composition of raw materials and products (g/100 g) [15].

Samples	Protein	Ash	Fat	Dietary fiber			
				Insoluble fraction	Including chitin	Soluble fraction	Total
BW	49.51^{i}±0.16*	4.71^{i}±0.03	26.44^{b}±0.22	12.96^{i}±0.04	7.33^{i}±0.01	0.00^{a}±0.00	12.96^{k}±0.04
CF	55.18^{j}±0.11	4.34^{h}±0.07	29.01^{d}±0.04	18.48^{j}±0.06	9.92^{h}±0.01	0.24^{b}±0.01	18.72^{l}±0.05
TM	45.39^{h}±0.06	3.86^{g}±0.01	14.29^{a}±0.06	11.56^{h}±0.07	6.93^{g}±0.02	0.36^{c}±0.02	11.92^{j}±0.05
CN	21.50^{g}±0.03	2.53^{f}±0.00	45.62^{i}±0.48	5.19^{d}±0.01	0.00^{a}±0.00	0.67^{e}±0.06	5.86^{e}±0.05
HN	16.64^{f}±0.08	2.15^{e}±0.01	64.31^{j}±0.37	9.58^{g}±0.08	0.00^{a}±0.00	1.49^{j}±0.00	11.07^{i}±0.08
ST	10.78^{a}±0.02	1.49^{a}±0.01	36.20^{h}±0.06	3.14^{a}±0.03	0.00^{a}±0.00	0.84^{g}±0.01	3.98^{a}±0.02
BW15	13.23^{b}±0.15	1.51^{a}±0.02	34.07^{f}±0.01	4.14^{b}±0.06	0.77^{b}±0.01	0.85^{g}±0.04	4.99^{b}±0.09
BW30	14.06^{d}±0.01	1.57^{c}±0.01	32.62^{e}±0.16	5.20^{d}±0.04	1.91^{e}±0.01	0.84^{g}±0.03	6.04^{f}±0.07
CF15	13.48^{c}±0.04	1.56^{b}±0.01	32.91^{e}±0.08	4.88^{c}±0.07	1.01^{c}±0.01	0.75^{f}±0.04	5.63^{d}±0.03
CF30	15.51^{e}±0.04	1.73^{d}±0.01	26.69^{c}±0.11	6.04^{f}±0.01	2.35^{f}±0.01	0.53^{d}±0.01	6.57^{h}±0.03
TM15	13.11^{b}±0.12	1.52^{a}±0.01	35.48^{g}±0.23	4.14^{b}±0.09	0.77^{b}±0.01	1.04^{i}±0.01	5.18^{c}±0.11
TM30	13.69^{c}±0.16	1.57^{c}±0.00	29.35^{d}±0.23	5.46^{e}±0.03	1.53^{d}±0.01	0.95^{h}±0.02	6.41^{g}±0.05

*Values in the same row marked with different letters are statistically significantly different at $p < 0.05$. BW – buffalo worm (*A. diaperinus*) flour, CF – cricket (*A. domesticus*) flour, TM – *T. molitor* flour (mealworm flour), S – standard bar, BW15, BW30 – bars with 15, 30 % addition of buffalo worm flour, CF15, CF30 - bars with 15, 30 % addition of cricket flour, TM15, TM30 - bars with 15, 30 % addition of *T. molitor* flour, CN – cashew nut, HN - hazelnuts.

Table S2. Amino acid profile of raw materials and enriched products [15].

Samples	Protein (%)	Essential amino acids (mg /g of protein)								Total EAA
		His	Ile	Leu	Lys	Met	Phe	Thr	Val	
BW	49.512^{i}±0.16	40.358^{h}±0.998	48.459^{cb}±1.127	74.781^{b}±1.963	73.166^{hg}±2.161	22.208^{bc}±2.679	48.761^{b}±1.874	44.982^{c}±1.018	64.196^{cd}±1.293	416.913^{c}±7.876
CF	55.181^{j}±0.11	25.854^{c}±0.168	44.194^{b}±0.263	79.143^{b}±0.581	57.258^{c}±0.649	19.696^{a}±0.851	37.108^{a}±0.311	41.039^{b}±0.427	62.401^{c}±0.764	366.935^{b}±2.373
TM	45.391^{h}±0.06	35.5528^{g}±0.467	47.384^{bc}±0.783	77.474^{b}±1.284	60.851^{dc}±0.983	20.138^{ab}±2.191	36.873^{a}±0.565	43.892^{c}±0.855	67.622^{de}±1.149	389.787^{b}±4.064
CH	21.503^{g}±0.03	18.788^{b}±0.560	32.348^{a}±0.888	57.833^{a}±1.446	39.553^{b}±0.858	23.703^{cb}±2.519	37.004^{a}±0.654	28.546^{a}±0.763	44.806^{b}±1.161	282.582^{a}±8.557
HN	16.642^{f}±0.08	21.634^{a}±0.657	31.373^{a}±1.171	57.229^{a}±1.929	24.392^{a}±0.747	26.303^{d}±1.369	37.445^{a}±1.293	26.582^{a}±0.763	40.131^{a}±1.378	265.089^{a}±7.362
ST	10.781^{a}±0.02	31.410^{ef}±3.001	63.457^{g}±6.004	109.665^{e}±10.549	68.818^{fg}±7.736	40.395^{g}±1.791	71.906^{f}±6.477	56.880^{g}±5.103	77.349^{ih}±6.955	519.880^{e}±44.118
BW15	13.233^{b}±0.15	30.117^{e}±0.327	56.426^{f}±0.362	94.714^{dc}±0.795	66.114^{ef}±2.609	36.996^{e}±1.051	61.727^{de}±0.514	50.782^{ed}±0.301	69.511^{efg}±0.305	466.387^{d}±3.836
BW30	14.064^{d}±0.01	33.212^{fe}±0.660	57.287^{f}±0.884	95.295^{dc}±1.590	69.464^{gh}±1.549	35.784^{f}±0.619	62.409^{ed}±2.511	52.931^{fe}±0.565	72.314^{fgh}±0.615	478.697^{d}±7.321
CF15	13.481^{c}±0.04	28.088^{dc}±0.409	55.803^{ed}±0.347	97.421^{d}±0.449	64.084^{e}±1.536	36.275^{f}±0.281	60.688^{de}±2.192	51.313^{edf}±0.257	70.967^{efg}±0.596	464.639^{d}±3.869
CF30	15.510^{e}±0.04	26.797^{cd}±0.362	52.702^{d}±1.094	91.128^{c}±1.659	60.047^{dc}±1.173	33.826^{e}±1.360	54.311^{c}±1.125	49.221^{d}±1.086	68.284^{ef}±1.503	436.316^{c}±7.866
TM15	13.113^{b}±0.12	30.885^{ef}±1.625	57.266^{f}±2.735	99.303^{d}±4.799	62.280^{dce}±4.493	34.616^{ef}±1.044	61.249^{de}±2.969	52.476^{fe}±2.592	73.187^{gh}±2.619	471.262^{d}±20.910
TM30	13.691^{c}±0.16	31.933^{ef}±2.287	57.299^{f}±3.666	98.398^{d}±6.459	66.508^{ef}±4.975	36.871^{f}±0.847	58.002^{cd}±3.608	54.029^{f}±2.859	74.253^{hgf}±4.399	477.293^{d}±28.552

*Values in the same row marked with different letters are statistically significantly different at $p < 0.05 \pm$ SD. EAA, essential amino acids. HN – hazelnut, CN – cashews nuts, CF – cricket *A. domesticus*, TM - mealworm *T. molitor*, BW - buffalo worm *A. diaperinus*.

Table S3. Fatty acids profile of nut bars raw materials and enriched products (g/100 g) [15].

Fatty acids	BW	CF	TM	CN	HN	ST	BW15	BW30	CF15	CF30	TM15	TM30
C16:0	27.211b± 0.019*	28.896a ±0.001	23.340c ±0.143	9.137f ±0.008	5.988l ±0.011	8.764g ±0.003	9.606e ±0.021	11.639d ±0.004	7.395k ±0.009	7.627j ±0.008	7.807i ±0.024	8.198h ±0.020
C18:0	8.553b ±0.009	11.855a ±0.041	4.683j ±0.037	8.252c ±0.042	3.065k ±0.031	5.597f ±0.006	5.716e ±0.004	5.034i ±0.010	5.626f ±0.004	5.322h ±0.002	5.970d ±0.013	5.432g ±0.008
C18:1 cis oleic	34.119i ±0.002	25.852j ±0.001	54.871h ±0.360	62.971g ±0.033	82.441a ±0.063	70.963d ±0.052	70.507e ±0.076	69.560f ±0.019	73.880b ±0.004	73.865b ±0.004	73.104c ±0.062	73.107c ±0.002
C18:2 (9.12) n-6 cis	24.843a ±0.022	23.364b ±0.054	7.804l ±0.042	17.703c ±0.009	7.866k ±0.004	12.746d ±0.018	12.346e ±0.009	10.934j ±0.005	11.726g ±0.001	11.812f ±0.000	11.063h ±0.023	11.003i ±0.021
C18:2 (9.12) n-6 trans	0.099b ±0.003	0.284a ±0.002	0.106b ±0.011	n.d.	n.d.	0.019d ±0.000	0.021cd ±0.001	0.028c ±0.000	0.014d ±0.003	0.021cd ±0.001	0.015d ±0.001	0.015d ±0.000
∑ SFA	37.763b ±0.014	40.751a ±0.078	28.023c ±0.284	17.389d ±0.007	9.053j ±0.042	14.361g ±0.014	15.322f ±0.040	16.673e ±0.016	13.020i ±0.008	12.949i ±0.008	13.777h ±0.042	13.630h ±0.015
∑ MUFA	34.119i ±0.016	25.852j ±0.036	54.871h ±0.314	62.971g ±0.016	82.441a ±0.063	70.963d ±0.034	70.507e ±0.062	69.560f ±0.017	73.880b ±0.006	73.865b ±0.003	73.104c ±0.057	73.107c ±0.002
∑ PUFA	24.942a ±0.031	23.648b ±0.042	7.909j ±0.030	17.703c ±0.009	7.866k ±0.021	12.765d ±0.018	12.367e ±0.020	10.942i ±0.001	11.740g ±0.003	11.833f ±0.005	11.077h ±0.016	11.018h ±0.014
∑PUFA/ ∑SFA	0.694h ±0.001	0.580j ±0.002	0.282k ±0.001	1.018a ±0.000	0.869c ±0.002	0.889d ±0.000	0.807e ±0.001	0.657i ±0.000	0.902c ±0.001	0.914b ±0.001	0.804g ±0.001	0.808f ±0.002

*Values in the same row marked with different letters are statistically significantly different at $p < 0.05$ ± SD. HN – hazelnut, CN – cashews nuts, CF – cricket *A. domesticus*, TM - mealworm *T. molitor*, BW - buffalo worm *A. diaperinus*.
